# Scattering from ‘Babinet’ particles (or not…): spherical particles made up of spheres and spherical particles with spherical voids

**DOI:** 10.1107/S1600576725010143

**Published:** 2026-02-01

**Authors:** Jan Skov Pedersen, Thea Lykkegaard Møller, Milena Corredig

**Affiliations:** a Department of Chemistry and Interdisciplinary Nanoscience Center (iNANO) Aarhus University Gustav Wieds Vej 14 Aarhus8000 C Denmark; b Department of Food Science, CiFOOD Center for Innovative Food Research Aarhus University Agro Food Park 48 Aarhus8200 N Denmark; Brazilian Synchrotron Light Laboratory, Brazil

**Keywords:** small-angle scattering, composite particles, internal structure, Monte Carlo simulations, analytical form factors, polydispersity

## Abstract

The small-angle scattering form factors of two classes of composite particles with contrasting internal architectures have been studied: one consisting of inclusions of smaller spheres embedded within a larger sphere, and the other comprising a solid sphere with randomly distributed spherical voids. These systems serve as material- and void-based analogues, providing a model framework for examining how internal material distribution in porous particles influences scattering signatures. Monte Carlo simulations were used to generate scattering curves across a range of volume fractions and polydispersities, which were then employed to benchmark analytically derived form factor expressions. Steric repulsion between, respectively, spheres and voids was taken as hard-sphere interactions. The results reveal that internal structural asymmetries, especially in spatial correlations and contrast topology, significantly affect scattering patterns, despite the particles having similar overall structures and volume fractions. In particular, spheres-of-spheres structures exhibit features in the scattering signal from internal modulations, while void-based particles display smoother shell-like scattering features. The analytical models show excellent agreement with the simulated data, capturing both the global shape and fine structural characteristics. These findings demonstrate that relatively simple analytical approaches, validated against numerical simulations, can reliably describe complex heterogeneous particles. This methodology provides a robust basis for interpreting scattering data from porous and composite materials across a wide range of applications.

## Abstract 2

Small-angle scattering form factors of composite particles built from either embedded spheres or randomly distributed voids within a sphere are obtained by Monte Carlo simulations, and analytical expressions that reproduce the simulations are derived.

## 1. Introduction

Complex composite particles are ubiquitous in nature, but studies of their internal architecture are limited [see, for example, Czajka *et al.* (2021 ▸), de Kruif (2014 ▸), Genix & Oberdisse (2013 ▸), Spinozzi *et al.* (2024 ▸), Tiihonen *et al.* (2024 ▸) and Jeong *et al.* (2012 ▸)]. The particles in these publications are, respectively, a polymer/silica nanocomposite, casein micelles in milk, fillers in rubber, lipid nanoparticles, polymer–carbon nanocomposites and double emulsions. Scattering from particles such as protein aggregates, colloidal complexes or structured micelles presents significant challenges due to their internal heterogeneity and the interplay between core–shell architectures, matrix inhomogeneities and polydispersity (Musino *et al.*, 2018 ▸; Larsen *et al.*, 2020 ▸; Spinozzi *et al.*, 2024 ▸). Previous reports describing scattering from aggregates include those for fractals (Teixeira, 1988 ▸; Dimon *et al.*, 1986 ▸) and random-flight aggregates (Burchard & Kajiwara, 1970 ▸), which are reviewed in detail by Larsen *et al.* (2020 ▸). More recently, theoretical developments such as models for casein micelles (Hansen *et al.*, 1996 ▸; Pedersen *et al.*, 2022 ▸) and the ‘dirty snowball’ model for heterogeneous microgels (Keerl *et al.*, 2009 ▸) have provided a powerful framework for interpreting small-angle scattering data from particles with internal structure. The approaches used in the calculations are related to those used in, for example, the derivation of block copolymer micellar models (Pedersen & Gerstenberg, 1996 ▸; Svaneborg & Pedersen, 2012*a* ▸,*b* ▸). Such modelling methodologies have been successfully applied to describing structural organization in protein biophysics (Larsen *et al.*, 2020 ▸; Rasmussen *et al.*, 2022 ▸; López Hernández *et al.*, 2025 ▸) and in applied biology, for example in food protein systems (Pedersen *et al.*, 2022 ▸; Li *et al.*, 2025 ▸), where hierarchical structures are abundant. In the latter cases, hierarchical composite models enabled a detailed interpretation of scattering profiles across multiple length scales. These examples highlight how robust well founded analytical models can be used to disentangle the scattering contributions from different internal regions within complex polydisperse particles.

A central question in small-angle scattering is whether different material configurations can give rise to in­distinguishable scattering patterns. This fundamental ambiguity arises because small-angle scattering is not directly sensitive to specific molecular identities or configurations, but rather to the spatial correlations of scattering contrast relative to a homogeneous background. As a result, distinct physical systems may produce similar or identical scattering signals when their contrast distributions exhibit statistically equivalent correlations. This is elegantly illustrated by the generalized Babinet principle [see *e.g.* Porod (1982 ▸)], which holds that the scattering from an object and from its complement, the negative space it occupies, can, under idealized conditions, be indistinguishable. In bulk systems, this principle implies that a homogeneous matrix containing randomly dispersed voids may, in some cases, yield the same scattering as a collection of dense particles dispersed in a medium, provided that the contrast is inverted and the spatial correlation functions are preserved.

The conceptual insight provided by the Babinet principle prompts a question in relation to the scattering derived from composite colloidal particles: can we distinguish a compact particle composed of many embedded spheres from a solid sphere containing internal voids? In the idealized case, where only the spatial correlation of contrast matters and where surface effects and other real-world deviations are neglected, these two systems may produce identical small-angle scattering patterns. However, real systems often violate this symmetry due to specific structural features, such as finite size and finite interface widths, polydispersity, or variations in internal connectivity.

**Figure 1:**
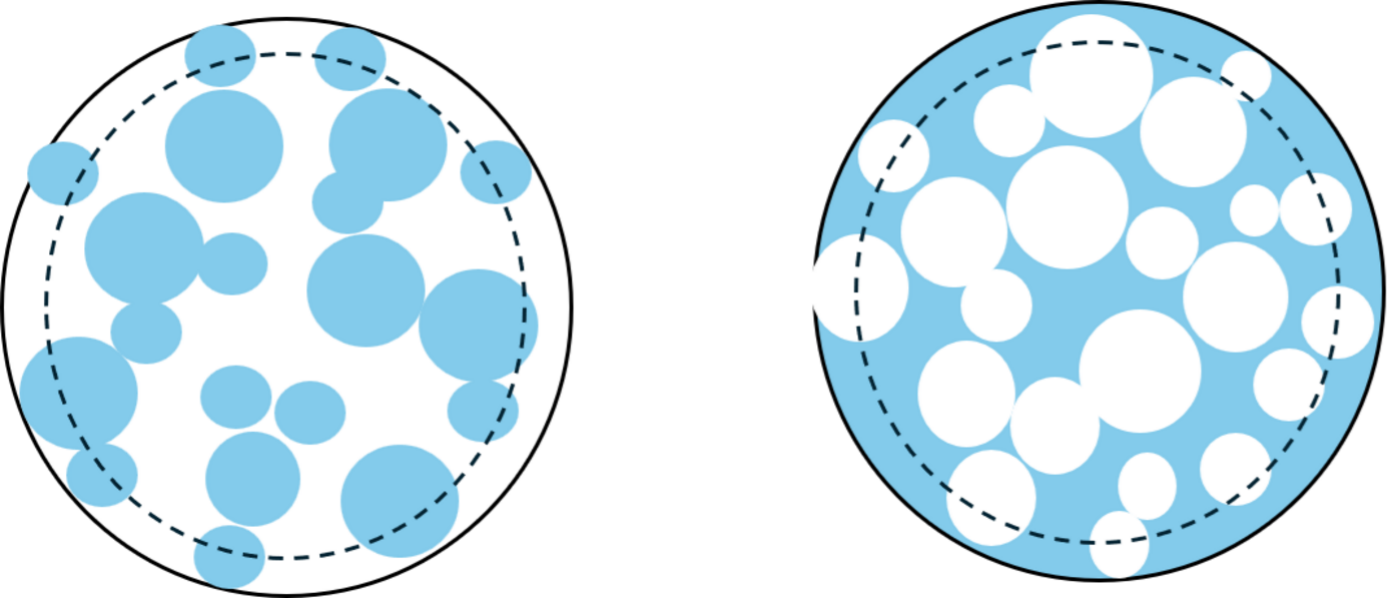
(Left) A sphere of spheres. (Right) A sphere with holes. The broken line indicates the outer radius limit for placing, respectively, the small spheres and the holes.

In this study, two types of composite particles, as illustrated in Fig. 1 ▸, are examined: one composed of smaller spheres embedded within a larger sphere and the other consisting of a solid sphere containing randomly distributed spherical voids or holes. Through this comparison, we will unravel how the underlying structural asymmetry manifests in measurable scattering differences. This will be done by performing Monte Carlo simulation methods on the two systems and generating their scattering form factors, varying both the volume fraction of material and the particles and polydispersities. In addition, analytical expressions to reproduce the simulation data will be derived and fitted to the simulation data.

## 2. Sphere of spheres with correlation between internal spheres

In this section, the scattering of a sphere of spheres (SoS) is calculated. The system consists of large polydisperse spheres of average radius *R*_L_ consisting of embedded small polydisperse spheres of average radius *R*_S_. The calculation of the form factor model proceeds in two steps. First, the form factor is calculated for one size of large spheres, and in the next step the polydispersity of these is taken into account. The number size distributions are assumed to follow a Schulz distribution. A list of the symbols used is given in Table 1 ▸.

The probability density function for the Schulz distribution for an average radius of *R* and a relative standard deviation of σ is
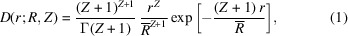
with *Z* = 1/σ^2^ − 1.

The large spheres are described by their average radius *R*_L_ and their relative standard deviation σ_L_, and the corresponding parameters for the small spheres are *R*_S_ and σ_S_. The centres of the small spheres are within a radius of *R*_L_ − *R*_S_ so that they only extend slightly outside the surface of a sphere with radius *R*_L_ (Fig. 1 ▸). The parameter η is the nominal volume fraction of small spheres for an infinitely large sphere, where the influence of boundaries is negligible.

The number of small spheres within a large sphere is calculated by first considering the average volume of a small sphere:
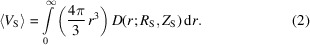


The number of spheres is then calculated as
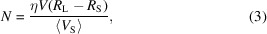
where *V*(*R*_L_ − *R*_S_) is the accessible volume for the small spheres: *V*(*R*_L_ − *R*_S_) = (4π/3)(*R*_L_ − *R*_S_)^3^.

The approach used in the following has been applied in a previous derivation of the ‘dirty snowball’ model (Keerl *et al.*, 2009 ▸) and more recently to the scattering of casein micelles from milk (Pedersen *et al.*, 2022 ▸).

The total intensity for monodisperse small particles is then
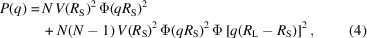
where Φ(*qr*) is the amplitude form factor of a sphere (Strutt, 1910 ▸) of radius *r*, *i.e.*
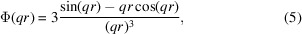
with the same definition of *V*(*X*) as above. The parameter *q* is the modulus of the scattering vector. The first term originates from particle self-interference and the second from interparticle interference. Note that *N* depends on the radius of the large sphere through *V*(*R*_L_ − *R*_S_).

When the particles are correlated due to high concentrations, this should be considered. As the first term is the only one which contributes at high values of *q*, interference effects can be included by modifying this term. This leads to
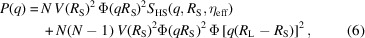
where the structure factor of hard spheres *S*_HS_(*q*, *R*, η_eff_) was chosen (Ashcroft & Lekner, 1966 ▸; Kinning & Thomas, 1984 ▸) as it has a simple analytical expression. The interaction further provides a simple approach for avoiding steric overlap of the spheres. Note that the analytical solution for the structure factor for monodisperse hard spheres in the Percus–Yevick approximation is only reliable up to a volume fraction of 0.4 for bulk systems and that it is also applied outside this range in the present work (Levesque & Verlet, 1969 ▸; Hansen & McDonald, 2013 ▸).

In this expression, η_eff_ is the effective volume fraction of small spheres within the large sphere. As some of them are at the border of the particle, they will have only about half as many neighbours as those in the centre, so it is reasonable to reduce the volume fraction to an effective value. The fraction of small spheres in the surface shell from *R*_L_ − 2*R*_S_ to *R*_L_ − *R*_S_ is
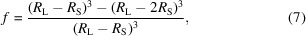
giving an effective volume fraction estimate of
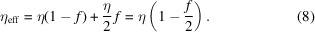


Next, polydispersity of the small spheres will be included in the expression. Let
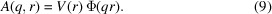
We then have the average form factor and amplitudes given by
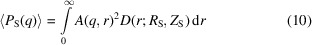
and we also need the average scattering amplitude, 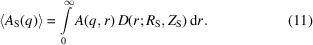


With this, the scattering for polydisperse small particles can be calculated as
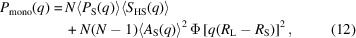
where 〈*S*_HS_(*q*)〉 is an effective structure factor.

Inspired by the scaling approximation (Gazzillo *et al.*, 1999 ▸), one may approximate the effective structure factor by
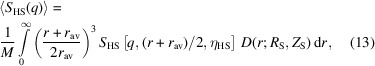
where 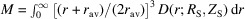 is a normalization factor and
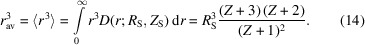
Note that this approximation, compared with the original scaling approximation, has the advantage that it only has a single integration over the size distribution and is therefore much faster in a numerical implementation.

In the final step one averages this form factor over the distribution of the large spheres,
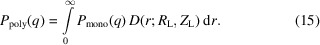
Although this can be used straightforwardly in a numerical implementation, a more efficient approach can be worked out. Using the relationship from before, 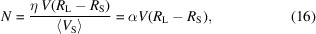
which defines α, and for
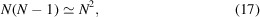
which is valid if *N* >> 1, we have
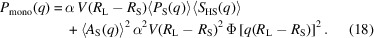
This means that the polydisperse form factor becomes
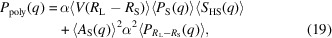
with an obvious definition of the 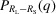 term. The analytical expression given by Pedersen *et al.* (2022 ▸) for the average form factors 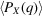 and amplitude 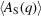 can then be used for fast calculations and only 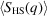 has to be calculated numerically.

## 3. Sphere with holes and correlations between holes

For the derivation of the scattering from sphere with holes (SwH) particles, one can reuse most of the above equations. However, the volume fraction needs to describe what is left in an infinitely large sphere when the holes are introduced.

The number of spheres/holes per large particle *N* is estimated by
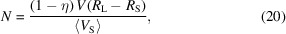
where η is the volume fraction of material after the holes are made. The effective volume fraction is estimated as
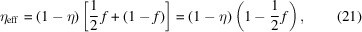
where *f* is calculated in the same way as before [*cf.* equation (7)].

The total intensity for monodisperse small particles is
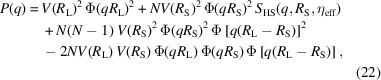
where a structure factor for describing correlations between holes is already included. For polydisperse small spheres using again an effective average structure factor, this becomes
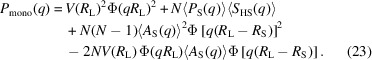


We then obtain the final expression which includes polydispersity of the large spheres, written in the same way as for the previous model by simply integrating over the size distribution:
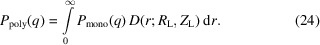


Note that the number of holes *N* also depends on *R*_L_. Using a similar relationship to before,
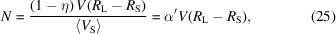
which defines α′ and gives
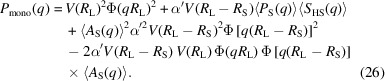


For calculations where there is polydispersity of the large spheres, the first three terms are easily integrated over the size distribution of the large spheres, whereas the last one is difficult to calculate analytically. Hence the averaging of this term is more easily performed numerically.

For remaining terms with 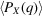 and 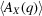, the analytical expressions from Pedersen *et al.* (2022 ▸) are used again, as they result in efficient and fast calculations. This gives formally
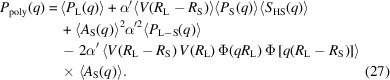
Here, only 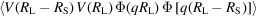 and 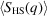 have to be calculated numerically. Note that, throughout these derivations, the excess scattering length density is neglected, since it is only one phase that scatters.

## 4. Monte Carlo simulations of scattering

The simulations were performed similarly to those described by Pedersen *et al.* (2022 ▸) in a two-stage process: first generating a configuration of small spheres, then using that configuration to model and compute scattering from larger composite spheres.

For low volume fractions of spheres and holes, the configurations were fast and easy to equilibrate. For larger volume fractions of spheres and holes, generation of an equilibrated configuration is much more difficult as the system tends to reach a jammed state. Therefore, the simulation begins by generating a dense packing of small hard spheres on a face-centred cubic lattice. Each sphere is initially assigned a radius equal to the average value of a target Schulz distribution, which characterizes the desired polydispersity. Over the course of the Monte Carlo simulation, the radii are gradually adjusted to their final values drawn from the Schulz distribution. This gradual transformation ensures that the system remains free of overlaps and reaches equilibrium smoothly. With this approach, it was possible for spheres with a polydispersity of 20% to reach equilibration without overlap for volume fractions η up to 0.6.

Note that when the radii of large and small spheres are generated, it is checked that the average and standard deviation are in agreement with the desired values, within 1%. This ensures that the selected distributions are representative of the limiting distribution for many spheres.

When overlaps are checked in the simulations, the minimum image convention (Allen & Tildesley, 2017 ▸) is used for the system with periodic boundary conditions. The system is equilibrated using standard Monte Carlo moves with a reasonable acceptance rate of 0.3–0.9, and configurations are sampled once the system has reached a steady state.

Once a well equilibrated configuration of small spheres is obtained, it is used as a building block to construct larger composite spheres. These large spheres are modelled as spherical volumes filled with the previously generated small-sphere configurations. Each large sphere contains a statistically representative subset of the small spheres, preserving the original size distribution and spatial correlations. Note that, in practice, the radii of the large spheres are generated first so that the box size can be chosen to be large enough for the largest radius of the large spheres. Simulations were then conducted differently depending on the volume fraction of the small particles. For the low volume fractions, the simulations were repeated ten times and each block was used for ten different large-sphere radii. For the large volume fractions, it took a much longer time to reach an equilibrated configuration, and therefore, to make the best use of the configuration, the scattering was calculated in a different way: First, the neighbouring cells were filled in using shifts of the original box and 50 different centre values were randomly generated within the central box. For each centre value, the scattering was then calculated for ten different large radii, giving a total of 500 different radii.

The scattering intensity was computed using the Debye equation (Debye, 1915 ▸), summing over all the spheres selected for a certain value of *R*_L_ and then subsequently averaging over *R*_L_ values as indicated by 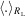:
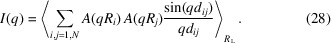
Here *d_ij_* is the distance between the centres of the small spheres.

The scattering from spheres with holes is calculated in a similar manner, except that the large spheres are included in the sum and the signs of the scattering amplitudes *A*(*qR*_*i*_) are negative for the small ones.

## 5. Results from Monte Carlo simulations

For systems with *R*_L_ = 100 Å, σ_L_ = 0.2 and *R*_S_ = 10 Å, σ_S_ = 0.2, simulations could be run for nominal volume fractions η up to 0.6 for the SoS configurations and, therefore, for volume fractions down to 0.4 for the SwH. A series of simulations for polydispersities σ_L_ = σ_S_, in the range 0.1 to 0.5 for constant η = 0.3 for the SoS and thus η = 0.7 for the SwH, were then carried out.

**Figure 2:**
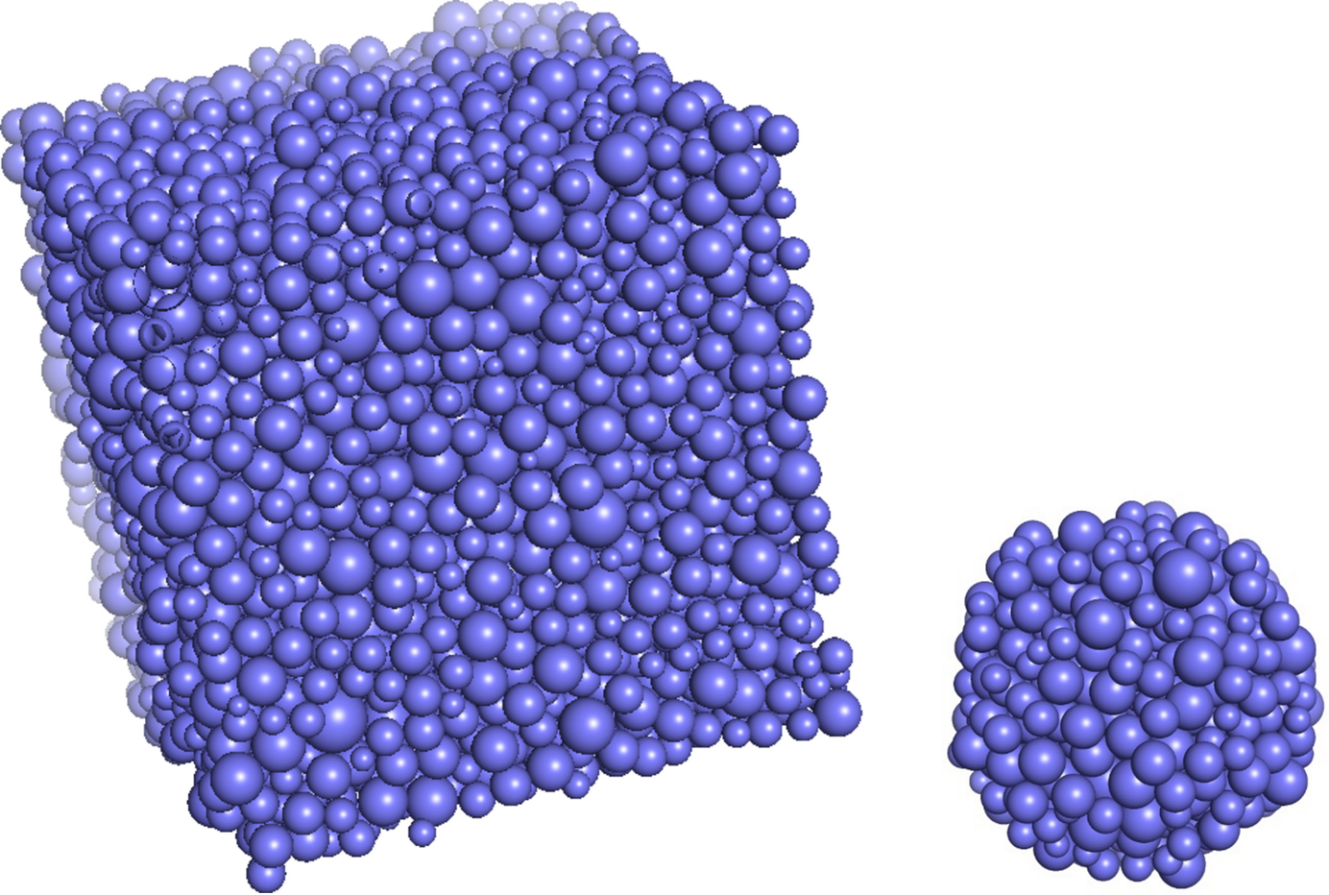
An example of an equilibrated structure for the full box to be used for *R*_L_ = 100 Å, σ_L_ = 0.2, and *R*_S_ = 10 Å, σ_S_ = 0.2 and η = 0.6, and also the selected small sphere constituting a sphere of radius 100 Å.

Fig. 2 ▸ shows an example of an equilibrated structure for the full box to be used for *R*_L_ = 100 Å, σ_L_ = 0.2 and *R*_S_ = 10 Å, σ_S_ = 0.2 and η = 0.6, and next to it is the selected small sphere for a large sphere with *R*_L_ = 100 Å.

**Figure 3:**
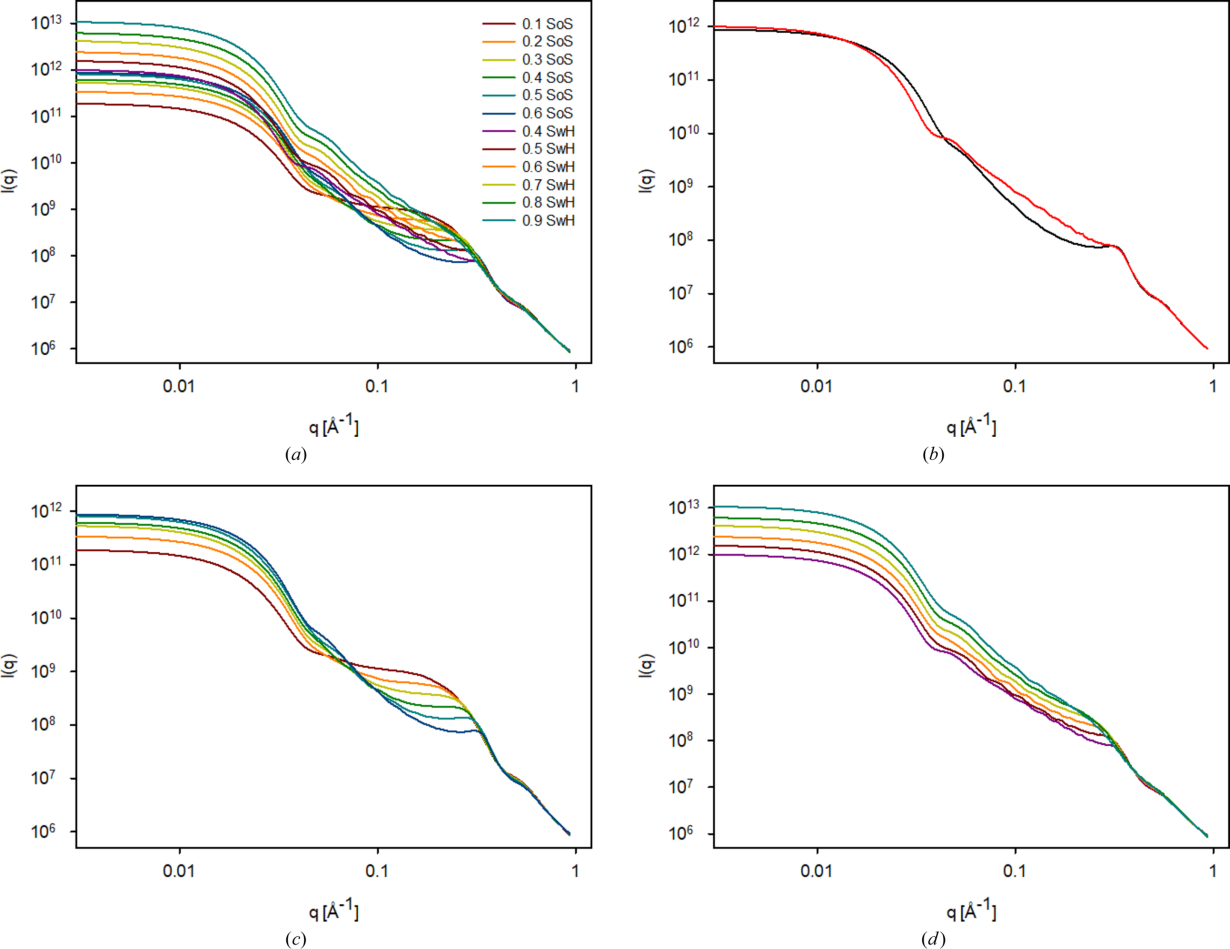
(*a*) All simulations for *R*_L_ = 100 Å, σ_L_ = 0.2 and *R*_S_ = 10 Å, σ_S_ = 0.2 for both a sphere of spheres and a sphere with holes. Nominal volume fractions are shown in the figure inset. (*b*) The two closest results for 0.6 for the SoS (black) and 0.4 for the SwH (red). (*c*) A comparison between SoS plots. From bottom to top at low *q*: η = 0.1, 0.2, 0.3, 0.4, 0.5 and 0.6. (*d*) A comparison between SwH plots. From bottom to top at low *q*: η = 0.4, 0.5, 0.6, 0.7, 0.8 and 0.9.

All results for *R*_L_ = 100 Å, σ_L_ = 0.2 and *R*_S_ = 10 Å, σ_S_ = 0.2 with various nominal volume fractions are shown in Fig. 3 ▸. The results are scaled to agree at high *q*. The curves show surprisingly smoothly changing behaviour. The plot is somewhat crowded for the middle volume fractions as both types of particles have scattering in this region. The two simulations with the closest actual volume fractions within the spheres are shown in Fig. 3 ▸(*b*). One sees that the SwH has a larger radius of gyration as the curve crosses over to a constant value at a lower *q* than for the SoS. This is due to the SwH having more material at the surface and thus being slightly shell-like.

For the data for the SoS, the scattering from the internal spheres is directly observed at intermediate and high *q* (>0.08 Å^−1^) [Fig. 3 ▸(*c*)], whereas it is somewhat more hidden for the SwH [Fig. 3 ▸(*d*)] as the surface of the larger sphere containing the holes contributes significantly to the scattering in this case. The internal correlations, which are reflected in the structure factor, are also clearly visible for the SoS for *q* > 0.2 Å^−1^, but the structure factor results in just a small bump for the SwH. In fact, for the SwH, the scattering decay shows almost a *q*^−2^ dependence in the middle- to high-*q* range (0.06–0.3 Å^−1^) for the particles with the highest volume fraction of holes (η = 0.4). This *q* dependence is similar to the scattering from a shell structure.

The main difference when comparing the two configurations is that the results for the SwH reflect that this composite particle is slightly shell-like, with a higher density at the edge. This gives rise to a larger radius of gyration and a levelling off at a lower *q* value than the SoS configuration. The data shown in Fig. 3 ▸ also clearly demonstrate that the radius of gyration increases with an increase in the number of holes in the particle, as the shell-like structure gradually develops with the presence of more holes. In contrast, the SoS has a constant radius of gyration, independent of the number of spheres present within the particle.

For the SwH in Fig. 3 ▸, there is an intermediate region with a power-law behaviour with an exponent between −3 and −2, which could lead to the consideration of whether the particles could be fractal. However, it is only a very narrow crossover region in *q* and it originates from a combination of surface Porod scattering and the slightly shell-like structure of the particles, as the holes are not allowed to penetrate the surface of the larger sphere. Fractals are also not expected since the procedure used for generating the bulk structures should not give rise to fractals, which are generated by entirely different step-by-step aggregation in diffusion-limited or reaction-limited aggregation (Anitas, 2020 ▸).

**Figure 4:**
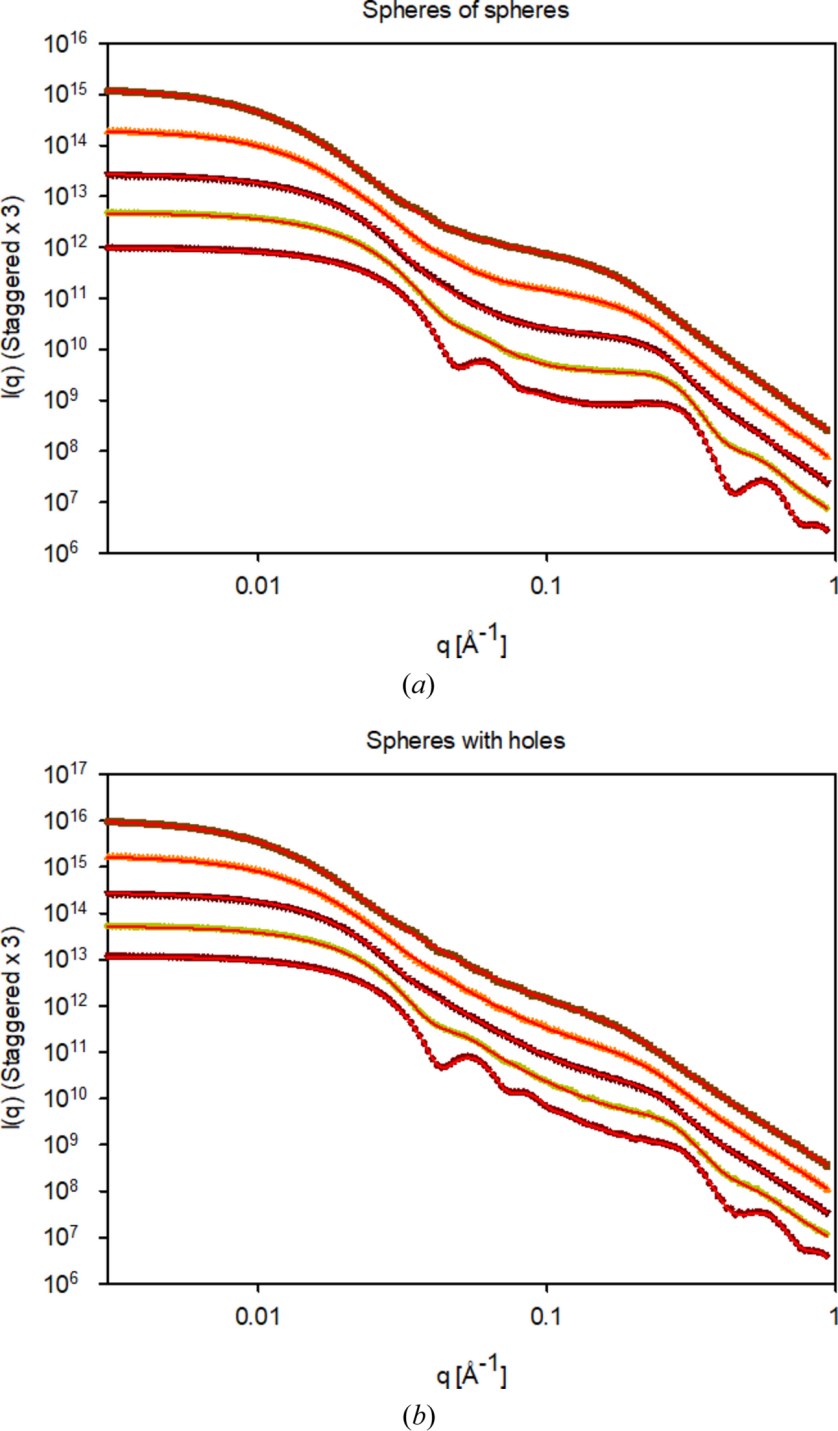
Varying polydispersity of both large and small spheres (0.1, 0.2, 0.3, 0.4 and 0.5 – bottom to top) for (*a*) spheres of spheres and (*b*) spheres with holes for a large sphere of overall size with the same polydispersity. Scattering was calculated for internal spheres with a volume fraction fixed at 0.3 for the spheres of spheres and at 0.7 for the spheres with holes. The red curves are the fits to the simulation data using the analytical expressions. Plots of *I*(*q*) are staggered.

Fig. 4 ▸ displays the series of simulations for polydispersities (σ_L_) in the range between 0.1 and 0.5 for a constant η = 0.3 for the SoS, and conversely η = 0.7 for the SwH.

## 6. Analysis of the simulated data using the theoretical expressions

The simulation data were fitted using the theoretical expressions derived above. To have some flexibility to fit the data, an overall scale was included, as well as scale factors for the hard-sphere radius *S*_*R*HS_ and the effective volume fraction *S*_ηHS_ entering the structure factor. This is a reasonable practice, since the expression used for the effective structure factor is only an approximation; furthermore, the hard-sphere structure factor is not expected to be reliable in the monodisperse case, when the volume fraction is larger than 0.4. Fitting *S*_*R*HS_ and *S*_ηHS_ then allows for correcting effective volume fraction estimates and average interaction radius values. Note that the nominal volume fraction entering the expressions determines the number of small particles within the large spheres, and it therefore gives the ratio of the forward scattering to the scattering at high *q* generated from the small particles. Therefore, the nominal volume fraction η was optimized. Besides these parameters, *R*_L_, σ_L_, *R*_S_ and σ_S_ were also optimized. This is reasonable considering the inherent uncertainty of the simulations due to random variations.

**Figure 5:**
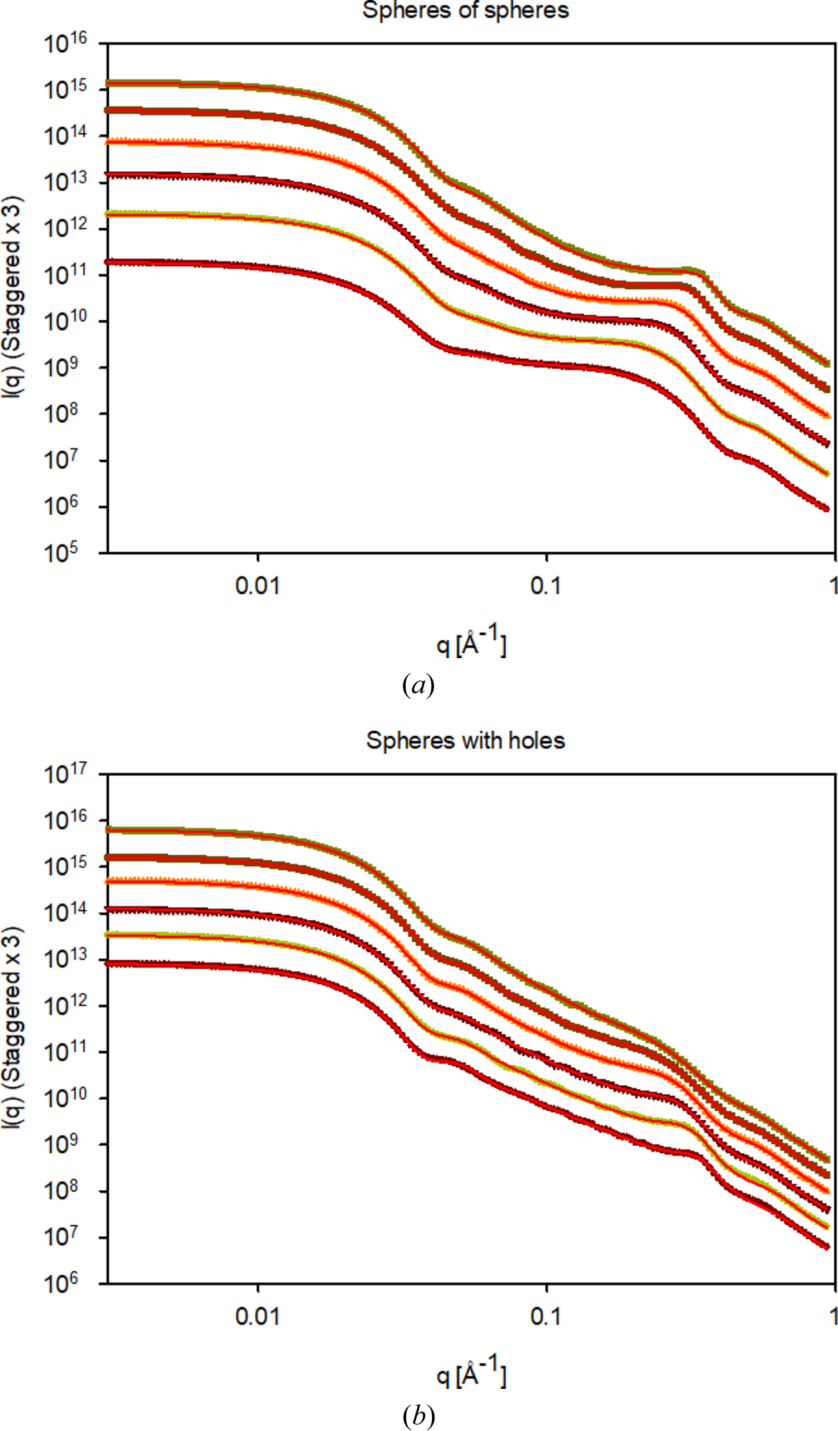
Fits to the simulation data in Fig. 3 using the analytical models for varying volume fractions and *R*_L_ = 100 Å, σ_L_ = 0.2 and *R*_S_ = 10 Å, σ_S_ = 0.2. (*a*) SoS for volume fractions (bottom to top) η = 0.1, 0.2, 0.3, 0.4, 0.5 and 0.6. (*b*) SwH for volume fractions (bottom to top) η = 0.4, 0.5, 0.6, 0.7, 0.8 and 0.9. Plots of *I*(*q*) are staggered.

Results from fitting the analytical model to the simulation data are summarized in Table 2 ▸ and the fits are shown in Fig. 5 ▸. The fits agree very well with the simulation data. Overall, there is good agreement between the results from the fits and the input values in the simulations. Exceptions are noted in Table 2 ▸ for the two highest volume fractions for the SoS, which are underestimated. As η determines the forward scattering, this is compensated by an increase in the scale factor. The scales in the structure factor are within ±10% of unity, except for the SoS at a high volume fraction, for which *S*_ηHS_ is about 20% lower than unity, and for the SwH containing a large density of holes, where *S*_*R*HS_ is about 20% lower than unity. These results suggest a surprising accuracy of the approximation used for the structure factor at a polydispersity of 0.2, up to very high volume fractions for the underlying Monte Carlo simulations: 60% spheres and 60% holes, respectively.

The simulation data resulting from constant volume fractions but varying polydispersity were also fitted by the analytical models. Comparisons between simulations and fits are shown in Fig. 4 ▸, with results summarized in Table 3 ▸. Also for these data, the fits agree very well with the simulation data. All determined parameters agree very well with the input parameters of the simulations, except for *S*_ηHS_ which decreases with increasing polydispersity, from unity at low values to around 0.6 for a polydispersity of 50%. This is somewhat expected, as approximations for the structure factor will overestimate the effects at high polydispersities (Pedersen, 1994 ▸; Gazzillo *et al.*, 1999 ▸).

## 7. Summary and conclusions

This study has explored the scattering behaviour of two types of composite particles with contrasting internal structures: one where smaller spheres form a larger particle and the other being a solid sphere containing randomly distributed spherical voids. These two architectures represent granular particles and void-containing analogues, respectively, and offer a useful framework for examining how internal material distribution in composite particles influences small-angle scattering curves.

Monte Carlo simulations were employed to generate scattering curves for composite particles with these different internal architectures. The simulated data served as benchmarks for evaluating the accuracy of analytically derived expressions for the form factor. By systematically varying volume fractions and polydispersities, the simulations revealed how differences in spatial correlations and contrast topology influence the resulting scattering patterns. This approach allowed us to test and validate the analytical models under controlled conditions, showing how internal organization can lead to distinct features in the scattering curves, even when the overall contrast volume remains similar.

The internal structural differences are clearly manifest at lower and intermediate *q*, with notable effects on the overall radius of gyration and the presence or absence of modulations associated with internal correlations. Systems with a granular structure exhibited more pronounced internal features, while those with internal voids often produced smoother profiles resembling those of shell-like particles with a high volume fraction of voids.

Analytical models were derived to reproduce the simulated scattering patterns and showed excellent agreement across a wide range of parameters. These models successfully captured both the shape of the scattering curves and the underlying structural trends, demonstrating that relatively simple analytical descriptions can yield accurate interpretations even in complex polydisperse systems. Note that it is the form factor scattering that has been derived, which means that is has implicitly been assumed that there are no interparticle structure factor effects. For future applications where this condition is not fulfilled due to longer-range interactions and/or concentration effects, such effects have to be considered. As the overall scattering amplitude of the particles is not readily available from the presented theoretical expressions, we suggest that one includes the structure factor effects in the local monodisperse approximation (Pedersen, 1994 ▸), since it does not require the amplitudes.

Overall, the comparison highlights how internal structural asymmetries, whether composed of a granular structure or a material with voids, play a central role in shaping scattering behaviour. The methods presented here can readily be extended to a variety of other structurally complex systems of composite nanoparticles. These include porous colloidal particles, gel-filled microcapsules, lipid vesicles containing internal inclusions, biomolecular assemblies such as virus capsids with encapsulated cargo and lipid nanoparticles carrying bioactives. The methods could also be applied to engineered nanostructures such as hollow silica spheres or block-copolymer particles with internal domains. By adapting the internal geometry and contrast profiles, the same Monte Carlo and analytical framework can provide valuable insights into the structure–scattering relationship across a broad class of hierarchical and composite materials, although the extensions mentioned here are significantly more complex to handle in both the simulations and the analytical calculations.

While the particles themselves may resemble ‘Babinet pairs’, the overall scattering system does not fulfil the Babinet principle, since the particles in the pair are assumed to be surrounded by the same matrix or solvent. The systems are interesting and unusual in that the internal structure of the particles is inverted, allowing the full range of internal density to be explored. However, even at 50% volume fraction, the two types of particles are not identical and therefore do not fulfil the Babinet principle to produce indistinguishable scattering curves. This deviation can be attributed to their finite size. In conclusion, the results for these particles do not contradict the Babinet principle in small-angle scattering.

## Figures and Tables

**Table 1:** List of symbols

Symbol	Description
*D*(*r*; *R*, *Z*)	Schulz distribution with average *R* and *Z* = 1/σ^2^ − 1, where σ is the standard deviation
*R*_S_	Average radius of small sphere
σ_S_	Standard deviation of distribution of small spheres
*R*_L_	Average radius of large sphere
σ_L_	Standard deviation of distribution of large spheres
*V*(*R*)	Volume of sphere with radius *R*
*N*	Number of small spheres, or holes in large sphere
*q*	Modulus of scattering vector
Φ(*x*)	Scattering amplitude of sphere
*S*_HS_(*q*, *R*, η)	Structure factor of monodisperse hard spheres with radius *R* and volume fraction η
η_eff_	Effective volume fraction within large particles
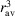	Cube of the radius related to the average volume of small spheres
*f*	Fraction of spheres or holes in the surface layer.
*P*_S_(*q*), *A*_S_(*q*)	Form factor and scattering amplitude for small spheres, weighted with, respectively, square of volume and volume of sphere.
*P*_L_(*q*)	Form factor for large spheres, weighted with square of volume
α = η/〈*V*_S_〉	Ratio between volume fraction and average volume of small spheres
α′ = (1 − η)/〈*V*_S_〉	Ratio between unity minus volume fraction and average volume of small spheres (holes)
*S*_*R*HS_	Scale of hard-sphere radius when fitting the simulated scattering data for the structure factor
*S*_ηHS_	Scale of hard-sphere volume fraction when fitting the simulated scattering data for the structure factor

**Table:** The first column is the nominal input volume fraction in the simulations. s.d. is the standard deviation.Spheres of spheres.

η	Scale	s.d.	*R*_L_	s.d	σ_L_	s.d.	*R*_S_	s.d.	σ_S_	s.d.	η	s.d.	*S*_*R*HS_	s.d.	*S*_ηHS_	s.d.
0.1	0.97	0.01	100.7	0.2	0.197	0.002	9.91	0.03	0.207	0.001	0.103	0.001	0.953	0.011	0.91	0.02
0.2	1.06	0.02	97.6	0.4	0.216	0.003	10.05	0.05	0.201	0.003	0.206	0.002	1.018	0.009	1.10	0.03
0.3	1.00	0.01	99.3	0.2	0.205	0.002	9.96	0.03	0.189	0.001	0.296	0.002	0.964	0.004	0.97	0.01
0.4	1.04	0.02	101.4	0.3	0.187	0.002	9.92	0.04	0.189	0.002	0.382	0.004	0.934	0.005	0.97	0.01
0.5	1.14	0.03	102.3	0.4	0.189	0.003	9.94	0.05	0.177	0.003	0.456	0.006	0.910	0.005	0.97	0.02
0.6	1.31	0.05	99.6	0.7	0.213	0.006	9.85	0.06	0.160	0.004	0.518	0.010	0.874	0.005	0.95	0.03

**Table:** Spheres with holes.

η	Scale	s.d.	*R*_L_	s.d	σ_L_	s.d.	*R*_S_	s.d.	σ_S_	s.d.	η	s.d.	*S*_*R*HS_	s.d.	*S*_ηHS_	s.d.
0.4	1.05	0.02	99.4	0.6	0.212	0.005	9.56	0.08	0.182	0.006	0.400	0.004	0.817	0.019	0.89	0.01
0.5	1.00	0.01	101.6	0.3	0.191	0.003	9.77	0.06	0.189	0.004	0.497	0.003	0.875	0.016	0.92	0.01
0.6	1.00	0.01	100.2	0.3	0.194	0.003	9.85	0.06	0.190	0.004	0.599	0.003	0.917	0.021	0.94	0.01
0.7	1.01	0.01	98.9	0.2	0.206	0.002	9.95	0.04	0.192	0.003	0.699	0.002	0.955	0.020	0.96	0.01
0.8	1.03	0.01	99.2	0.3	0.213	0.003	10.13	0.08	0.203	0.005	0.794	0.002	1.100	0.050	0.99	0.02
0.9	0.98	0.01	100.6	0.4	0.204	0.003	9.98	0.15	0.203	0.009	0.898	0.003	0.956	0.209	0.93	0.08

**Table:** The first column is the nominal input volume fraction in the simulations. s.d. is the standard deviation.Spheres of spheres.

η	Scale	s.d.	*R*_L_	s.d	σ_L_	s.d.	*R*_S_	s.d.	σ_S_	s.d.	η	s.d.	*S*_*R*HS_	s.d.	*S*_ηHS_	s.d.
0.3	1.07	0.01	99.7	0.1	0.116	0.001	10.05	0.01	0.102	0.001	0.287	0.001	0.95	0.01	1.00	0.01
0.3	1.14	0.01	99.2	0.2	0.226	0.002	10.10	0.03	0.203	0.002	0.281	0.001	0.95	0.01	0.96	0.01
0.3	1.13	0.04	102.3	0.6	0.301	0.004	9.94	0.18	0.328	0.010	0.276	0.005	0.98	0.02	0.90	0.05
0.3	1.18	0.07	97.1	1.0	0.445	0.006	9.55	0.32	0.425	0.018	0.283	0.008	1.00	0.03	0.72	0.07
0.3	0.94	0.06	106.3	1.0	0.487	0.005	9.12	0.30	0.526	0.014	0.281	0.009	1.12	0.04	0.61	0.07

**Table:** Spheres with holes.

η	Scale	s.d.	*R*_L_	s.d	σ_L_	s.d.	*R*_S_	s.d.	σ_S_	s.d.	η	s.d.	*S*_*R*HS_	s.d.	*S*_ηHS_	s.d.
0.7	1.01	0.00	98.9	0.1	0.106	0.001	10.02	0.02	0.100	0.001	0.690	0.001	0.97	0.01	0.97	0.01
0.7	1.02	0.01	99.4	0.3	0.208	0.002	9.95	0.05	0.212	0.004	0.690	0.002	0.99	0.01	0.91	0.02
0.7	0.94	0.01	104.4	0.3	0.316	0.003	9.59	0.09	0.353	0.004	0.706	0.002	1.06	0.01	0.91	0.02
0.7	0.99	0.02	102.1	0.9	0.396	0.008	9.46	0.23	0.405	0.016	0.702	0.006	1.02	0.03	0.64	0.05
0.7	0.86	0.02	111.4	1.0	0.461	0.010	9.78	0.33	0.489	0.020	0.711	0.008	1.06	0.04	0.63	0.06
